# Multisymptom Burden in Cancer Survivors: Benefits of Physical Activity

**DOI:** 10.1249/esm.0000000000000029

**Published:** 2024

**Authors:** Thomas D. Cardaci, Brandon N. VanderVeen, Brooke M. Bullard, James A. Carson, E. Angela Murphy

**Affiliations:** 1 Department of Pathology, Microbiology, and Immunology, School of Medicine, University of South Carolina, Columbia, SC, USA;; 2 Department of Kinesiology and Sports Management, Sydney and J.L. Huffines Institute for Sports Medicine and Human Performance, Texas A&M University, College Station, TX, USA

**Keywords:** cachexia, cancer-related fatigue, cardiotoxicity, exercise, mucositis

## Abstract

In contrast with other leading causes of mortality, the cancer death rate in the United States continues to decline, reflecting improvements in prevention, screening, and treatment. Despite these advances, there has been limited development of strategies to counter the unwanted and debilitating effects associated with cancer and its treatments. Indeed, syndromes including cachexia, cardiotoxicity, fatigue, and mucositis among others plague cancer survivors, leading to poor life quality and premature mortality. The systemic nature of these impairments creates a strong rationale for treatment strategies to mitigate syndromes affecting cancer survivors. Currently, however, there are limited treatments approved by the US Food and Drug Administration to counter the debilitating side effects of cancer and cancer treatments. In noncancer clinical populations, physical activity is a well-established strategy to increase muscle mass, improve cardiovascular health, enhance energy levels, and promote gut health. Although physical activity programs are widely encouraged for cancer survivors, researchers are just beginning to understand the physiological basis of their positive effects and how they can be maximized for different cancer populations and treatments. This graphical review describes the benefits of physical activity and associated mechanisms for ameliorating select side effects of cancer and its therapeutics.

## INTRODUCTION

Cancer death rates have declined for all race groups over the last decade. Advances in cancer therapeutics including the emergence of immunotherapeutic approaches and improvements in radiation and chemotherapies have contributed to this increase in survival. Although treating the underlying malignancy remains at the forefront of anticancer efforts, understanding and mitigating the symptom burden associated with cancer and its therapeutics is critical to long-term cancer survivorship (1). Although many acute side effects can be adequately managed during cancer therapy (e.g., febrile neutropenia, acute nausea, and vomiting) and will resolve once therapy is complete, other adverse sequelae persist after therapy completion, lasting months or even years, and there are limited effective management strategies (1–4). Cachexia, cardiotoxicity, fatigue, and mucositis are common symptoms that plague cancer patients. Indeed, 50%–80% of cancer survivors suffer from cachexia (2), 37% have increased cardiovascular event risk, 52% have increased stroke risk (5), 80%–90% suffer from fatigue depending on the treatment regime (chemotherapy or radiation) (6), and up to 100% may experience mucositis (7). These adverse sequelae afflict cancer patients and survivors leading to poor life quality and even premature mortality (1–4). The complexity of these conditions is compounded by the fact that cancer survivors can experience six to nine symptoms at the same time (1). Symptoms may have synergistic effects, worsening other symptoms and ultimately leading to low life quality. For instance, neuropathy, exercise intolerance, cognitive dysfunction, sleep disturbances, anxiety, depression, and osteoporosis may occur independently from or secondary to cachexia, cardiotoxicity, fatigue, and mucositis. Symptom severity is critical to improved survival because a higher level of symptom burden has the potential to disproportionately impair overall function and life quality. Currently, there are few treatments approved by the US Food and Drug Administration (FDA) to counter these adverse events and those that are available are underutilized (e.g., dexrazozane for cardiotoxicity) and, consequently, many cancer patients must deal with the unknown ramifications of these effects.

**Figure 1.**
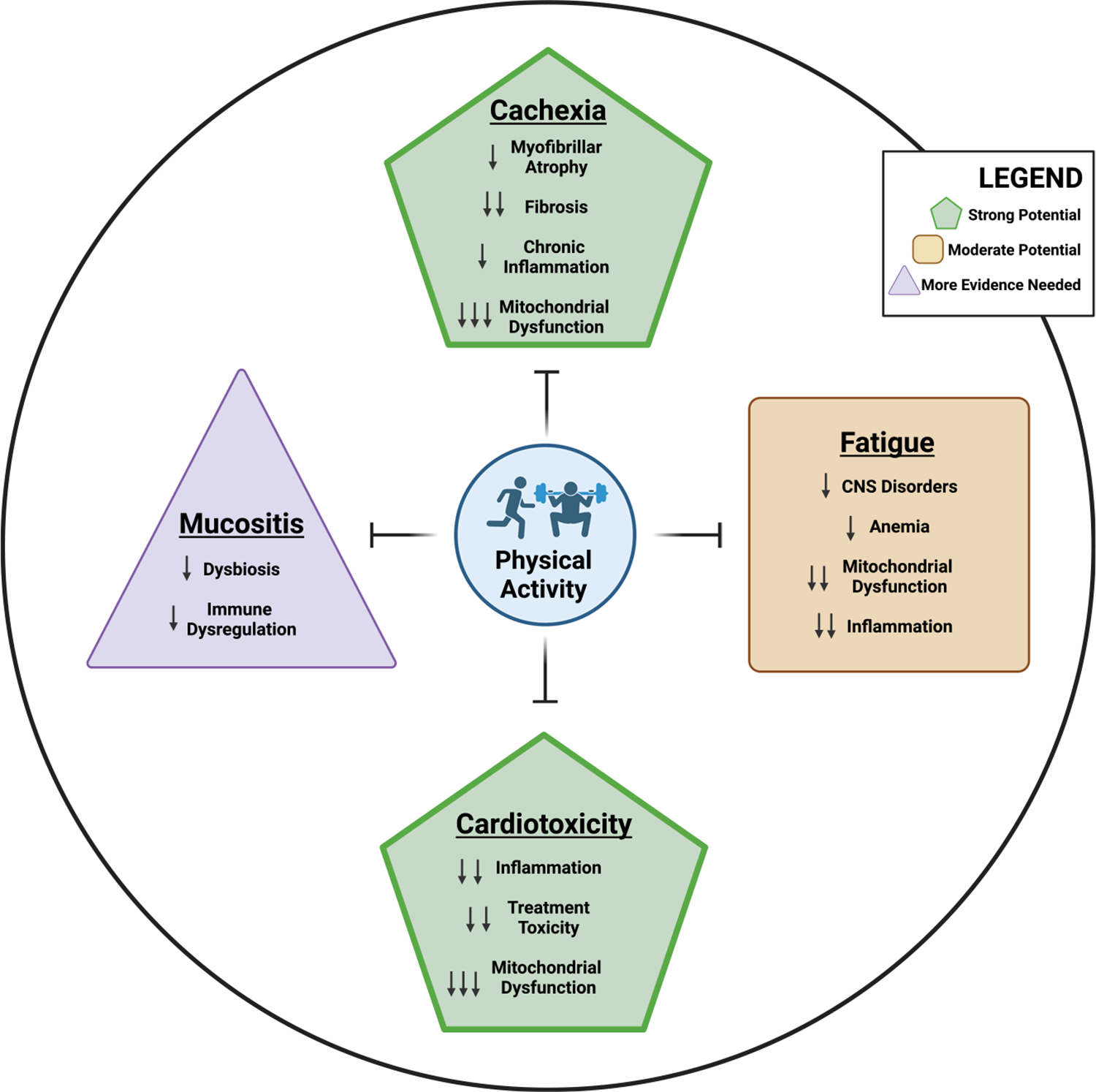
Benefits of physical activity on the adverse sequelae of cancer and its related treatments. The potential of physical activity to improve these cancer-related conditions is based on the strength of evidence in the literature. The number of arrows depicts the strength of evidence that physical activity improves the characteristics of the condition. CNS, central nervous system.

Physical activity is a well-established, safe, cost-effective, nonpharmacological strategy to increase muscle mass, enhance cardiovascular capacity, improve energy levels, and promote gut health in individuals without cancer. The multisystem benefits of physical activity or structured exercise provide a strong rationale for its implementation as a modality to mitigate the unwanted effects of cancer and its therapies in cancer survivors (Fig. 1). Indeed, the American College of Sports Medicine convened a roundtable in 2018 to advance physical activity recommendations beyond public health guidelines and toward prescriptive programs specific to cancer type, treatment, and outcome (8). Specifically, evidence-based frequency, intensity, time, and type (FITT) prescriptions with sufficient evidence were recommended for cancer survivors, acknowledging that more research is needed to fill the remaining gaps in knowledge to better serve cancer survivors and their healthcare providers (8). Unfortunately, although recommended, structured exercise is not widely implemented in standard care treatment for cancer patients. Reported barriers include treatment-related side effects, lack of time, fatigue, and insufficient education on the benefits of physical activity (9). In this graphical review, we 1) describe the adverse events associated with cancer treatments, 2) discuss the benefits of physical activity and/or structured exercise for alleviating these adverse events, and 3) provide suggestions throughout for future research to advance our understanding of the benefits of physical activity in cancer survivors. The focus of our review is on four critical cancer and cancer therapy side effects—cachexia, cardiotoxicity, fatigue, and mucositis—acknowledging that this is an incomplete list. Cancer patients can experience a multitude of other symptoms, which often occur in clusters, including neuropathy, exercise intolerance, cognitive dysfunction, sleep disturbances, anxiety, depression, and osteoporosis. However, a comprehensive discussion of all cancer-related symptoms is beyond the scope of this graphical review.

## CACHEXIA

Cancer cachexia is a multifactorial syndrome characterized by progressive and involuntary weight loss, muscle mass loss, and metabolic abnormalities (2,10). It is defined as a greater than 5% weight loss in 6 months that can be partially but not entirely reversed by conventional nutritional support (10). Strikingly, 50%–80% of cancer patients suffer from cachexia, which directly contributes to impaired treatment efficacy and tolerance, worsened functional capacity, diminished life quality, and even increased mortality (2). Specifically, the prevalence of cachexia is as high as 87% in patients with pancreatic and gastric cancers; 61% in prostate, lung, lymphoma, and colon cancers; and 40% in leukemia, sarcoma, and breast cancers (11). Although the etiology of cachexia is not well understood, it stems from the multifaceted interplay of secretion of tumor-associated inflammatory factors, metabolic alterations, and maladaptive host responses, culminating in severe skeletal muscle loss and dysfunction (e.g., myofibrillar protein loss, mitochondrial loss/dysfunction, fibrosis, myosteatosis, autophagy dysregulation) (9,12–15). For instance, elevations in tumor-secreted inflammatory cytokines such as interleukin (IL)-1β, IL-6, C-reactive protein (CRP), and tumor necrosis factor (TNF)-α trigger systemic inflammation and activation of skeletal muscle catabolic pathways (13). Specifically, cancer-induced activation of the ubiquitin–proteasome system and autophagy–lysosomal pathway activity drives myofibrillar protein degradation resulting in severe losses in muscle mass (13). Metabolic alterations, including disrupted glucose metabolism, excessive lipid deposition, or myosteatosis along with mitochondrial loss and dysfunction impair cellular energy production and further exacerbate muscle wasting (14). Concurrently, fibrosis, or excessive muscle collagen deposition, impairs muscle structural integrity and elasticity resulting in reduced contractility and overall function (15). Further, autophagy dysregulation also occurs in cachectic muscle reducing the muscle’s ability to remove dysfunctional cellular components and further contributing to impaired muscle function and mass regulation (13). These interconnected mechanisms create a relentless cycle of muscle degradation and metabolic disturbances, significantly impacting patient morbidity, physical function, and treatment efficacy.

**Figure 2.**
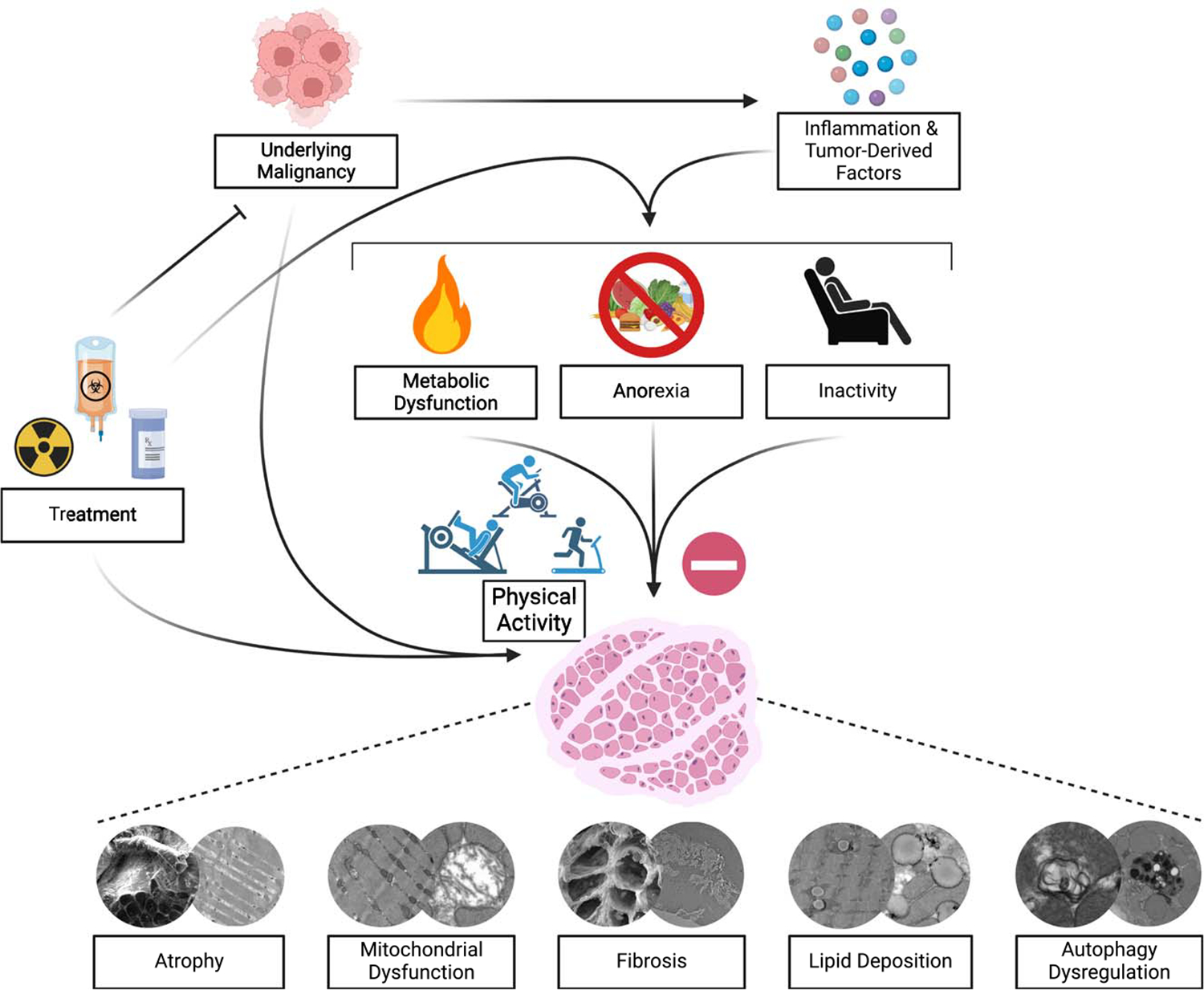
Impact of physical activity on ameliorating cancer cachexia and associated skeletal muscle dysfunction.

Despite the severity and prevalence of this condition, there are no standard treatments for cachexia; however, given the potent ability of physical activity and structured exercise to improve skeletal muscle health, augment immune function, and enhance metabolic health, it has emerged as a promising nonpharmacological therapeutic strategy to counteract cachexia and its associated symptoms (e.g., weakness, exercise intolerance, fatigue) (2,9). Preclinical studies highlight the benefits of exercise regardless of modality (i.e., resistance or aerobic) in multiple murine cachexia models, showing attenuation of cachexia progression (muscle loss, function loss, etc.) (12). Exercise’s ability to mitigate the progression of cachexia is thought to be attributed to its ability to counter increases in inflammatory factors, improve mitochondrial function, promote redox homeostasis, regulate muscle proteostasis, and augment metabolic health, among other things (Fig. 2) (2,9,12,16). Clinically, resistance exercise has been shown to improve upper and lower body function, preserve lean body mass, and mitigate cancer-induced increases in inflammatory factors without eliciting adverse effects in cancer patients (12,17). A recent meta-analysis including 13 human trials highlighted resistance exercise’s utility in elderly cancer patients wherein, regardless of cancer type or program duration (8–52 wk), resistance exercise increased muscle strength by 23% while preserving lean muscle mass (17). Endurance training has also been shown to be a safe and effective strategy to improve physical function in cancer patients by preserving skeletal muscle mitochondrial function, promoting redox balance, and reducing fatigue (12). Remarkably, even a single exercise session (resistance and/or aerobic exercise) has been shown to increase perceived energy by up to 24% and decrease self-reported stress by up to 33% and nausea by up to 79% in cancer patients undergoing chemotherapy treatment (18). Integrating both resistance and endurance exercise in a structured physical activity regimen may be optimal given the associated reductions in systemic inflammation, improvements in physical function, preservation of muscle mass, and bolstering of other psychosocial factors affecting cancer survivors (16,18). A clinical trial in prostate cancer patients undergoing androgen suppression therapy demonstrated increases in aerobic fitness, muscle strength, and lean mass with concomitant reductions in perceived fatigue and CRP following a 12-wk combined resistance and aerobic exercise intervention (16). In lieu of these benefits, the American College of Sports Medicine’s 2018 roundtable on physical activity guidelines for cancer survivors updated their recommendations to incorporate multiple exercise modalities (8).

Although the majority of clinical research supports the utility of physical activity, specifically structured exercise, for cancer patients and survivors in the context of mitigating cachexia, contention remains, likely due to the heterogeneity of cancer types, locations, stages, and symptoms; the variety and severity of cancer treatments; and the influence of other comorbidities (e.g., obesity, sarcopenia) (9). This is further complicated by biological age because the response to physical activity varies across the aging spectrum. Although a link between aging and cancer has been widely acknowledged, the risk of developing cancer, and by extension cachexia, is influenced by frailty status. These facts highlight the need for individualized and multimodal therapeutic approaches for cancer cachexia that incorporate both resistance and endurance exercise (9).

## CARDIOTOXICITY

Cancer patients and survivors are at an increased risk for major cardiovascular events. Cancer survivors have a 37% higher risk of developing cardiovascular disease (CVD) and a 52% higher risk of stroke (5). This can be best appreciated in adults who are childhood cancer survivors, given their increased risk for major cardiovascular events including myocardial infarction, heart failure, pericardial disease, valvular replacement, coronary artery disease, and/or serious arrhythmias later in life (19). The convergence of aging, sedentary lifestyle, cytotoxic chemotherapies, and radiation therapies contributes to CVD being the leading cause of death in cancer survivors (3).

Given the multitude of inputs, cardiotoxicity and cardiovascular events can occur similarly through multiple mechanisms. First, the increased risk of CVD with aging is vast and has been extensively reviewed and, therefore, is outside the scope of this review (20). Next, many of the most common cytotoxic chemotherapies, namely, anthracyclines, induce severe mitochondrial dysfunction and oxidative stress, resulting in myopericarditis and palpitations/tachycardia, with ~11% of patients experiencing severe toxicities following doxorubicin administration (21). Indeed, doxorubicin can accumulate within the mitochondria of cardiac tissue, perpetuating the impaired mitochondrial quality and increased oxidative stress. Additionally, many cancer patients, primarily with lung and breast cancers, undergo chest radiation therapy, which can directly damage the heart and surrounding tissues (22). Finally, as will be described later in this review, cancer patients and survivors suffer from debilitating fatigue, contributing to increased sedentary behavior, which negatively impacts cardiovascular health. Given the vast body of literature demonstrating physical activity and exercise’s ability to improve cardiorespiratory fitness with aging, improve cardiac mitochondrial function, and offset inactivity-induced cardiometabolic abnormalities, structured physical activity (i.e., exercise) is now being incorporated into cardio-oncology care.

Despite the significant impact of cancer, aging, and anticancer therapies on cardiorespiratory health, increasing physical activity and incorporating structured exercise hold the potential to either protect against the loss of cardiovascular health throughout treatment or improve cardiometabolic health in survivors. Several controlled clinical trials in breast cancer patients have recently been completed, and a range of effects have been demonstrated. First, Foulkes et al. (23) showed that, although structured exercise could not prevent functional decline with treatment, it prevented the loss of peak oxygen uptake $\left(\dot{\text{V}}{\text{O}}_{2\text{peak}}\right)$ and improved $\dot{\text{V}}{\text{O}}_{2\text{peak}}$ after 12 months. This was corroborated by Kerrigan et al. (24), who demonstrated that structured exercise protected against a loss in $\dot{\text{V}}{\text{O}}_{2\text{peak}}$ without evidence of adverse events. Unfortunately, not all trials have shown these same benefits and more work is certainly needed (9,25).

**Figure 3.**
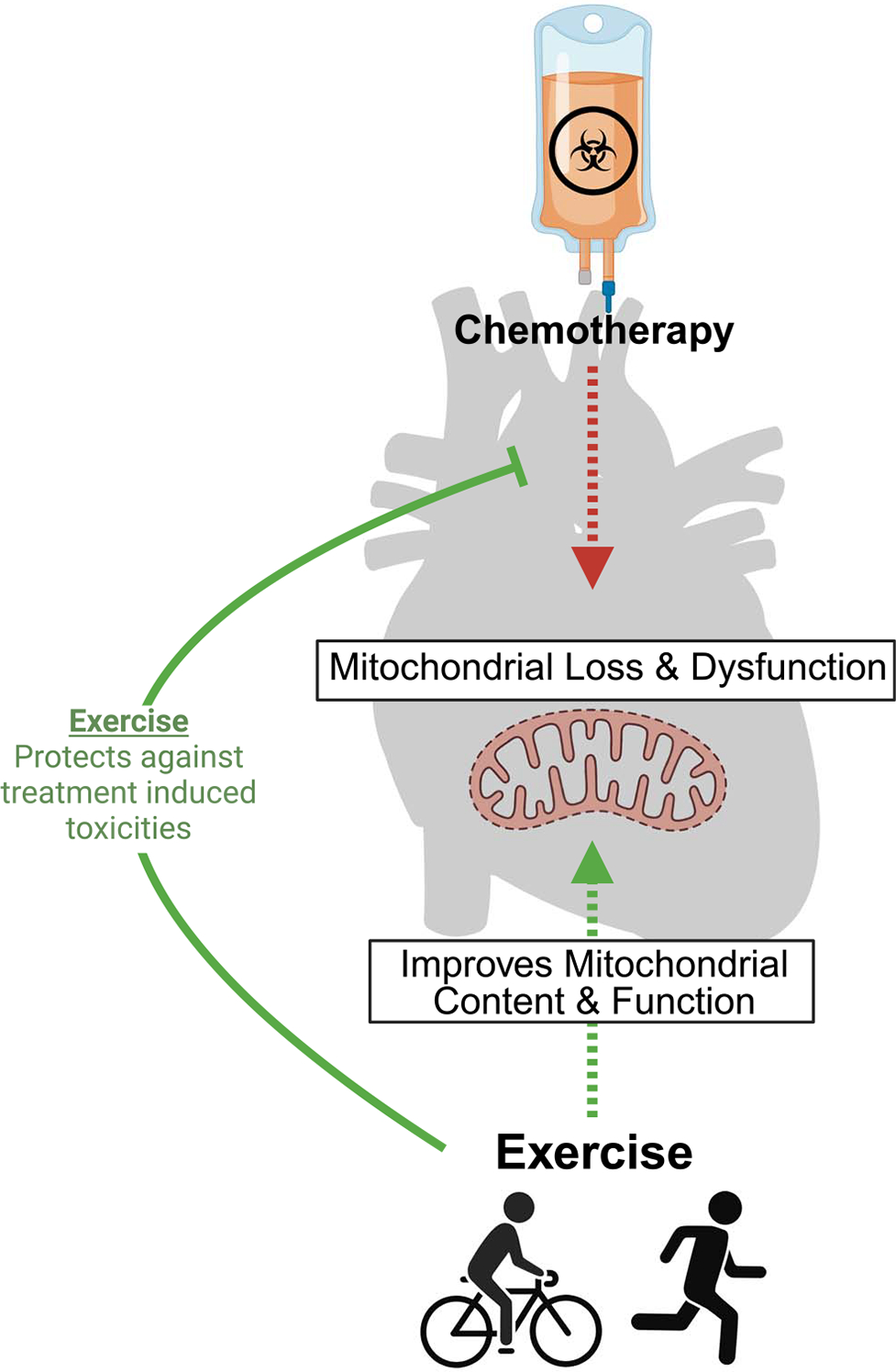
Physical activity counteracts cancer treatment–induced cardiotoxicity through improving skeletal muscle mitochondrial content and function.

The mechanisms by which aerobic physical activity improves cardiotoxicity with cancer are diverse and continue to be unearthed; however, decreasing the burden of chronic inflammation, promoting antioxidants, and improving mitochondrial content and quality continue to emerge as key outcomes for physical activity trials (Fig. 3) (3). A majority of studies in this domain have focused on the impact of aerobic activity as a cardioprotective intervention, which is the most commonly used training modality in cardiovascular care in cancer patients (26). Indeed, the available evidence suggests that both moderate- and high-intensity interval training are effective at preventing and treating CVD in cancer patients (26). Fewer studies have looked at the impact of resistance exercise on cardio-specific outcomes; nonetheless, it has been shown to be effective at improving muscular strength, muscle mass, and physical function as well as being safe and well tolerated for most patients with cancer (26). Combining endurance and resistance exercise will likely provide the most cardioprotective benefits given their synergistic effects on cardiorespiratory fitness, muscular strength, physical function, and body composition (26).

Prescribing physical activity across the cancer spectrum has demonstrated beneficial effects on cardiovascular fitness and prevents adverse cardiovascular events across several cancers and cancer therapies (26). However, the exact stimulus (i.e., FITT) required to prevent cardiovascular events in cancer patients remains unresolved and will likely require a personalized approach depending on baseline fitness, cancer type, treatment regime, and, by extension, exercise tolerance. Indeed, the 2018 American College of Sports Medicine roundtable was convened to advance physical activity recommendations toward prescriptive programs specific to cancer types, treatments, and/or outcomes (8). Interestingly, however, Scott et al. (27) demonstrated that the timing of exercise therapy had little impact on cardiorespiratory fitness improvements compared to usual care, suggesting that exercise should be prescribed at any time during the cancer continuum.

## FATIGUE

Cancer-related fatigue (CRF) is defined by the National Comprehensive Cancer Network as a distressing, persistent, subjective sense of physical, emotional, and/or cognitive tiredness or exhaustion related to cancer or cancer treatment that is not proportional to recent activity and interferes with usual functioning (4). CRF occurs as a consequence of the cancer itself and as a side effect of cancer treatment (6). Indeed, CRF may be an early symptom of malignant disease (40% of patients) or as a side effect of chemotherapy (80% of patients) or radiation (90% of patients) (6). CRF can reduce a patient’s ability to complete cancer treatments and participate in essential life activities, thus undermining quality of life and even reducing overall survival. It can continue for months and even years following completion of treatment (6). There is considerable variation in the severity and persistence of CRF among individuals, which is likely mediated by host factors, including characteristics that predate the cancer diagnosis as well as adverse physiological responses to cancer and its treatments (i.e., radiation and chemotherapy).

Despite the prevalence of CRF, the specific mechanisms involved in its pathophysiology have not been clearly defined (4). Proposed biological mechanisms that may drive CRF include the release of proinflammatory mediators, hypothalamic–pituitary–adrenal (HPA) axis dysregulation, circadian rhythm desynchronization, skeletal muscle wasting, and anemia (4); however, it is important to note that limited evidence supports these mechanisms. These gaps in understanding are likely driven by the facts that 1) there are no well-established animal models for studying CRF and 2) the complexity of this disorder, wherein many factors may be causative elements in the fatigue condition (e.g., pain, sleep disturbance, activity level, emotional distress). Nonetheless, there is possible evidence to support these plausible mechanisms. Inflammation has been widely associated with fatigue and other sickness behaviors, and it is well known that inflammation is a component of the tumor as well as a consequence of cancer treatments. Dysregulation in the HPA axis has been documented in CRF; indeed, CRF has been associated with increased evening cortisol levels and higher overall cortisol secretion (28). Cachexia, as described above, has been linked to CRF, given the associated asthenia (i.e., physical weakness, lack of energy). Finally, anemia, a consequence of cancer and its treatments, is a well-established mediator of fatigue and a likely contributor to CRF (29). Additional research is needed to establish the contribution of each of these mechanisms to CRF and then develop treatment approaches.

**Figure 4.**
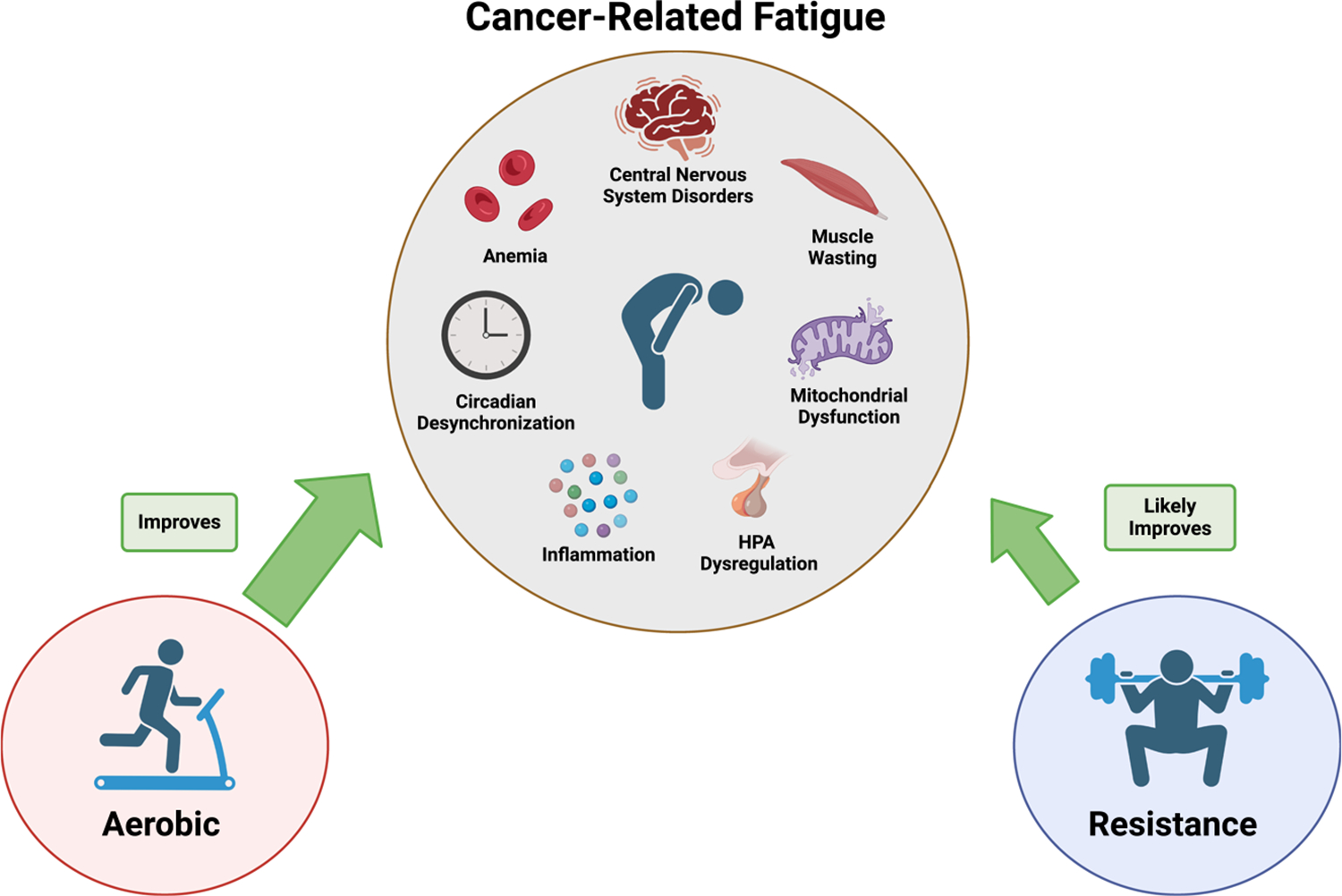
Effects of physical activity modalities on cancer-related fatigue and associated maladies. HPA, hypothalamic–pituitary–adrenal axis.

Although there are no FDA-approved treatments for CRF, clinical practice guidelines for its management exist; however, which mode of treatment is most effective remains elusive. Physical activity has emerged as a modality that holds great promise for mitigating CRF given its potential to target the presumed multifactorial mechanisms driving this syndrome. Several recent meta-analyses concluded that physical activity interventions effectively reduced CRF during and after cancer treatment and even more so than available pharmaceutical options (30,31). Indeed, a comprehensive meta-analysis including 110 well-designed randomized controlled trials reported that treatments incorporating a physical activity component were effective in improving CRF, whereas pharmaceutical interventions were not (30). Although high heterogeneity has been documented in activity mode, a subgroup analysis revealed that aerobic activity appeared to be the most effective for reducing CRF (31); however, that might be more related to the acceptability of this type of activity among cancer patients (i.e., lower intensity and fewer adverse events) rather than efficacy, and the fact that there are simply fewer investigations on the impact of resistance exercise on CRF to draw firm conclusions. Nonetheless, it is well established that aerobic activity can improve the factors presumed to be driving CRF including proinflammatory responses, HPA axis dysregulation, circadian rhythm desynchronization, muscle wasting, and anemia (Fig. 4). Interestingly, engaging in physical activity three times per week was the most effective frequency for enhancing life quality (30), which is consistent with the 2018 American College of Sports Medicine roundtable consensus statement (8). Considering intensity and duration, research suggests that physical activity interventions involving moderately intense (55%–75% of heart rate maximum) aerobic activity ranging from 10 to 90 min are consistently effective at managing CRF (32).

Although guidelines for physical activity prescriptions to relieve CRF exist, the optimal approach likely depends on individual factors (e.g., type of cancer and treatment, fatigue intensity, activity tolerance). Further, and unfortunately, most clinical studies to date have relied exclusively on self-reports to document CRF, with few assessing the efficacy of physical activity interventions on potential biomarkers (e.g., inflammation and anemia) of CRF. The lack of reliable animal models to study CRF further compounds our mechanistic understanding of optimal or effective physical activity prescriptions. Nonetheless, physical activity holds great promise as an intervention to alleviate CRF, but a personalized approach and an improved understanding of the involved mechanisms will likely be required to maximize benefits.

## MUCOSITIS/DYSBIOSIS

**Figure 5.**
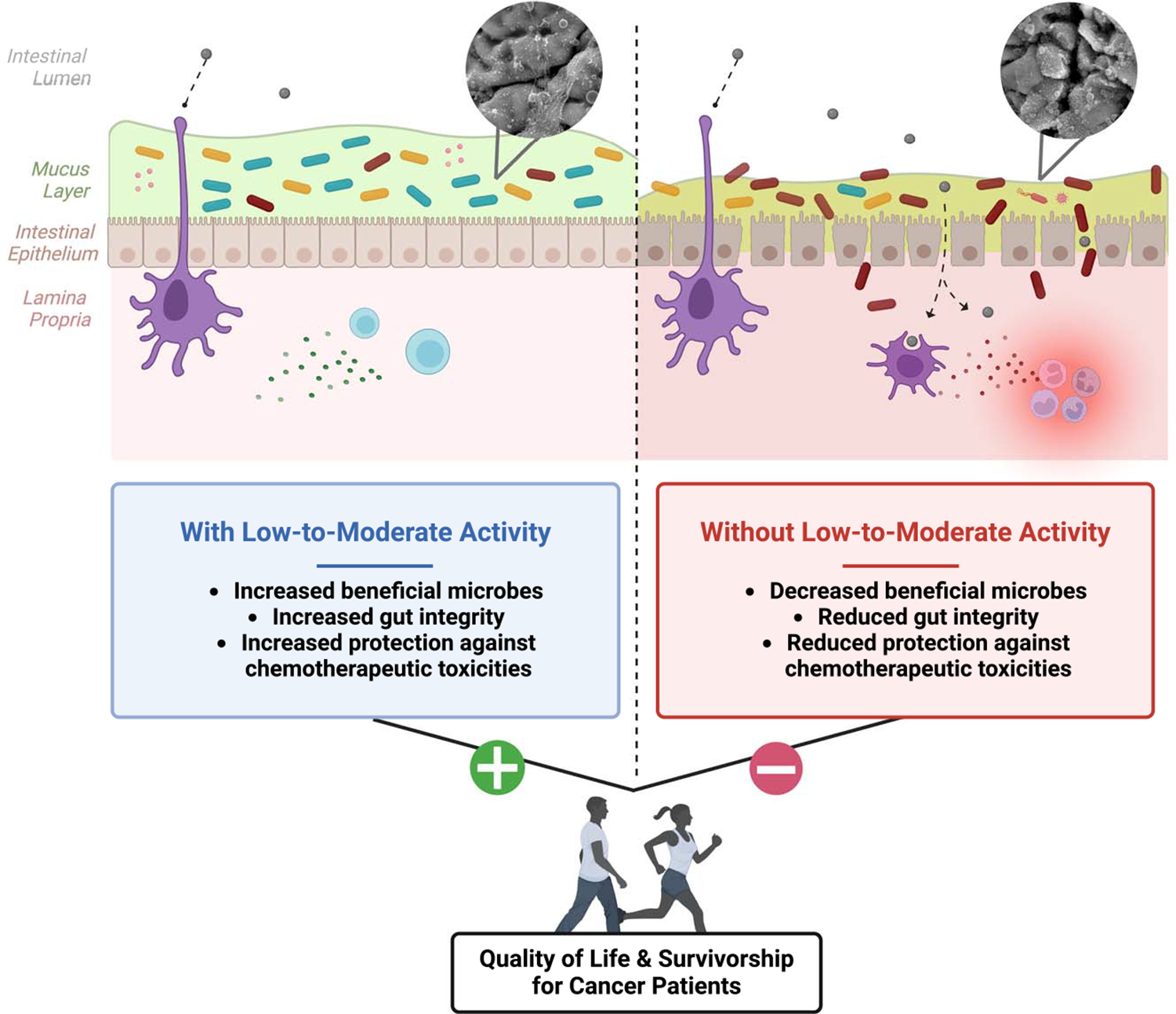
Potential benefits of physical activity on cancer- and cancer treatment–induced mucositis and dysbiosis.

Several cancers, most notably gastrointestinal (GI) cancers (e.g., pancreatic, colorectal, esophageal), are often diagnosed following patient-reported GI symptoms (e.g., pain, constipation or diarrhea, weight loss). Additionally, nausea, emesis (i.e., vomiting), diarrhea, mouth sores, painful swallowing, intestinal ulcerations, and abdominal pain are among the most reported side effects of cancer treatments. These off-target consequences contribute to anorexia, treatment dose reductions, reduced life quality, and increased mortality. One prevalent chemotherapy-induced perturbation is mucositis, which includes mucosal damage, inflammation of GI tissues (muscularis, lamina propria, etc.), and other gut-related side effects, and remains largely untreatable. Although considerable work has been done to understand and treat the side effects of mucositis, including the FDA approval of Zofran (i.e., ondansetron hydrochloride) for cancer-related nausea and vomiting, there are no FDA-approved drugs on the market to treat mucositis directly. As the etiology of mucositis continues to evolve, there appears to be a strong role for gut microbiota. Indeed, gut microbes can exert profound control over host physiology, including the immune response, mucosal layer, cellular repair mechanisms, and intestinal barrier integrity (33), which can all impact mucositis (Fig. 5). Gut microbiota has been recently reported to have a critical role in modulating the efficacy and toxicity of cancer immunotherapies and chemotherapeutics (33,34). For instance, a dysfunctional microbiota (i.e., dysbiosis) can disturb the metabolism of certain cancer drugs, resulting in altered circulating levels of toxic metabolites and increased exposure to carcinogens, which exacerbates mucositis and reduces survival (33,34).

Current literature supports the notion that physical activity can modulate gut microbes and consequently improve patient outcomes in the context of cancer and cancer therapy, with the most encouraging improvements observed with colorectal, breast, and prostate cancers (34,35). In fact, it has been reported, albeit in noncancer patients, that cardiovascular fitness can account for more than 20% of variation and diversity found in gut microbes (36). In the context of cancer, a preclinical study reported that low-intensity physical activity (i.e., walking) improved the microbiota profile following abdominal irradiation, which was consistent with reductions in inflammatory mediators and oxidative stress, ultimately attenuating radiation-induced toxicities (37). More specifically, low-intensity physical activity alleviated GI injury (i.e., improved histology and inflammatory gene expression in the intestines) and increased levels of *Akkermansia muciniphila* (*A. muciniphila*), which has been shown to have beneficial effects on intestinal stem cells, protect against intestinal inflammation, improve gut integrity, and potentially contribute to improved outcomes for cancer patients (37). In the same study, following irradiation and *A. muciniphila* supplementation, exercised mice had improved outcomes, including preserved colon lengths and reduced inflammation and reactive oxygen species in the intestines (37). In a 12-month cycling intervention for at-risk cancer patients, decreased colon inflammation, improved colonic mucosa, and enhanced cytotoxic immune response (i.e., natural killer cells and cytotoxic T-cells) were reported (38). Together, these findings support low- to moderate-intensity physical activity’s ability to improve the gut microbiota profile, which may improve inflammatory responses, reduce GI injury, and enhance survivorship in cancer patients.

Although the spectrum by which the gut responds to physical activity is still only vaguely understood and defined, there are a few possible mechanisms, including improved microbial diversity and metabolite production. Indeed, gut microbial diversity has been positively correlated with increased cardiorespiratory fitness in breast cancer survivors (39). Additionally, individuals who are physically active and have increased cardiorespiratory fitness have shown reduced levels of Bacteroidetes, and increased levels of Firmicutes, which is a phylum responsible (in part) for short-chain fatty acid (SCFA) production in the gut (33). SCFAs are vital sources of energy with an important role in maintaining balance between the host immune system and the intestinal microenvironment, and may play an important role in anticancer immunity. However, based on current published research, further investigation and additional human clinical trials are needed to fully understand the relationship between physical activity and gut health in the context of cancer survivorship. For example, one study has shown that alterations in gut microbes with cancer therapy can confer detrimental effects on physical activity outcomes (40). Indeed, grip strength and run time to fatigue were reduced following fecal microbiome transplantation from chemotherapy-treated mice (40). Thus, it is important to consider the bidirectional effects of the microbiota–physical activity relationship in the context of cancer survivorship. It appears that low-to-moderate aerobic physical activity regimens can potentially improve the colonic environment in cancer patients; however, the literature is currently lacking in this area to widely recommend resistance exercise for improving gut perturbations with cancer. As such, more research is required to provide a greater understanding of the influence of physical activity modality, intensity, and duration on the colon environment in the context of improving cancer survivorship. Moreover, mechanistic studies are needed to imply causation.

## CONCLUSION

Physical activity and exercise programs hold great promise for mitigating the symptom burden associated with cancer and its therapies. An American College of Sports Medicine roundtable was convened in 2018 to advance physical activity recommendations from those provided in 2010 toward prescriptive programs specific to cancer types, treatments, and/or outcomes (8). Although this signified the progress of the field, it also identified areas needing further research to fill the remaining knowledge gaps to better serve cancer survivors and their healthcare providers, ultimately improving clinical practice (8). In addition, further research is warranted, including preclinical mechanistic studies in animals, to understand the physiological basis for the benefits of physical activity on cancer-related symptoms.

## References

[1] Xiao C The state of science in the study of cancer symptom clusters. Eur J Oncol Nurs. 2010;14(5):417–34. doi:10.1016/j.ejon.2010.05.011. 20599421

[2] Leal LG, Lopes MA, Peres SB, Batista ML Jr. Exercise training as therapeutic approach in cancer cachexia: a review of potential anti-inflammatory effect on muscle wasting. Front Physiol. 2020;11:570170. doi:10.3389/fphys.2020.570170. 33613297 PMC7890241

[3] Viamonte SG, Joaquim AV, Alves AJ, Cardio-oncology rehabilitation for cancer survivors with high cardiovascular risk: a randomized clinical trial. JAMA Cardiol. 2023;8(12):1119–28. doi:10.1001/jamacardio.2023.3558. 37819656 PMC10568446

[4] Berger AM, Mooney K, Alvarez-Perez A, Cancer-related fatigue, version 2.2015. J Natl Compr Canc Netw. 2015;13(8):1012–39. doi:10.6004/jnccn.2015.0122. 26285247 PMC5499710

[5] Stoltzfus KC, Zhang Y, Sturgeon K, Fatal heart disease among cancer patients. Nat Commun. 2020;11(1):2011. doi:10.1038/s41467-020-15639-5. 32332714 PMC7181822

[6] Hofman M, Ryan JL, Figueroa-Moseley CD, Cancer-related fatigue: the scale of the problem. Oncologist. 2007;12(Suppl 1):4–10. doi:10.1634/theoncologist.12-S1-4. 17573451

[7] Peterson DE, Bensadoun R-J, Roila F, ESMO Guidelines Working Group. Management of oral and gastrointestinal mucositis: ESMO Clinical Practice Guidelines. Ann Oncol. 2011;22 Suppl 6(Suppl 6):vi78–84. doi:10.1093/annonc/mdr391. 21908510 PMC3662500

[8] Campbell KL, Winters-Stone KM, Wiskemann J, Exercise guidelines for cancer survivors: consensus statement from international multidisciplinary roundtable. Med Sci Sports Exerc. 2019;51(11):2375–90. doi:10.1249/MSS.0000000000002116. 31626055 PMC8576825

[9] Fairman CM, Lønbro S, Cardaci TD, Muscle wasting in cancer: opportunities and challenges for exercise in clinical cancer trials. JCSM Rapid Commun. 2022;5(1):52–67. doi:10.1002/rco2.56. 36118249 PMC9481195

[10] Fearon K, Strasser F, Anker SD, Definition and classification of cancer cachexia: an international consensus. Lancet Oncol. 2011;12(5):489–95. doi:10.1016/S1470-2045(10)70218-7. 21296615

[11] Dewys WD, Begg C, Lavin PT, Prognostic effect of weight loss prior to chemotherapy in cancer patients. Eastern Cooperative Oncology Group. Am J Med. 1980;69(4):491–7. doi:10.1016/s0149-2918(05)80001-3. 7424938

[12] Tsitkanou S, Murach KA, Washington TA, Greene NP. Exercise counteracts the deleterious effects of cancer cachexia. Cancers (Basel). 2022;14(10):2512. doi:10.3390/cancers14102512. 35626116 PMC9139714

[13] Zhang Y, Wang J, Wang X, The autophagic–lysosomal and ubiquitin proteasome systems are simultaneously activated in the skeletal muscle of gastric cancer patients with cachexia. Am J Clin Nutr. 2020;111(3):570–9. doi:10.1093/ajcn/nqz347. 31968072

[14] Stephens NA, Skipworth RJ, Macdonald AJ, Intramyocellular lipid droplets increase with progression of cachexia in cancer patients. J Cachexia Sarcopenia Muscle. 2011;2(2):111–7. doi:10.1007/s13539-011-0030-x. 21766057 PMC3117997

[15] Judge SM, Nosacka RL, Delitto D, Skeletal muscle fibrosis in pancreatic cancer patients with respect to survival. JNCI Cancer Spectr. 2018;2(3): pky043. doi:10.1093/jncics/pky043. 30637373 PMC6322478

[16] Galvao DA, Taaffe DR, Spry N, Combined resistance and aerobic exercise program reverses muscle loss in men undergoing androgen suppression therapy for prostate cancer without bone metastases: a randomized controlled trial. J Clin Oncol. 2010;28(2):340–7. doi:10.1200/JCO.2009.23.2488. 19949016

[17] Lee J. The effects of resistance training on muscular strength and hypertrophy in elderly cancer patients: a systematic review and meta-analysis. J Sport Health Sci. 2022;11(2):194–201. doi:10.1016/j.jshs.2021.02.002. 33592324 PMC9068528

[18] Johnsson A, Demmelmaier I, Sjovall K, A single exercise session improves side-effects of chemotherapy in women with breast cancer: an observational study. BMC Cancer. 2019;19(1):1073. doi:10.1186/s12885-019-6310-0. 31703567 PMC6842202

[19] Jones LW, Liu Q, Armstrong GT, Exercise and risk of major cardiovascular events in adult survivors of childhood hodgkin lymphoma: a report from the childhood cancer survivor study. J Clin Oncol. 2014;32(32):3643–50. doi:10.1200/JCO.2014.56.7511. 25311213 PMC4220043

[20] Paneni F, Diaz Canestro C, Libby P, The aging cardiovascular system: understanding it at the cellular and clinical levels. J Am Coll Cardiol. 2017;69(15):1952–67. doi:10.1016/j.jacc.2017.01.064. 28408026

[21] Chatterjee K, Zhang J, Honbo N, Karliner JS. Doxorubicin cardiomyopathy. Cardiology. 2010;115(2):155–62. doi:10.1159/000265166. 20016174 PMC2848530

[22] Belzile-Dugas E, Eisenberg MJ. Radiation-induced cardiovascular disease: review of an underrecognized pathology. J Am Heart Assoc. 2021;10(18): e021686. doi:10.1161/JAHA.121.021686. 34482706 PMC8649542

[23] Foulkes SJ, Howden EJ, Haykowsky MJ, Exercise for the prevention of anthracycline-induced functional disability and cardiac dysfunction: the BREXIT study. Circulation. 2023;147(7):532–45. doi:10.1161/CIRCULATIONAHA.122.062814. 36342348

[24] Kerrigan DJ, Reddy M, Walker EM, Cardiac rehabilitation improves fitness in patients with subclinical markers of cardiotoxicity while receiving chemotherapy: a randomized controlled study. J Cardiopulm Rehabil Prev. 2023;43(2):129–34. doi:10.1097/HCR.0000000000000719. 35940850

[25] Kirkham AA, Mackey JR, Thompson RB, TITAN trial: a randomized controlled trial of a cardiac rehabilitation care model in breast cancer. JACC Adv. 2023;2(6):100424. doi:10.1016/j.jacadv.2023.100424. 38939428 PMC11198667

[26] Wilson RL, Christopher CN, Yang EH, Incorporating exercise training into cardio-oncology care: current evidence and opportunities: *JACC: CardioOncology* state-of-the-art review. JACC CardioOncol. 2023;5(5):553–69. doi:10.1016/j.jaccao.2023.08.008. 37969654 PMC10635898

[27] Scott JM, Lee J, Herndon JE, Timing of exercise therapy when initiating adjuvant chemotherapy for breast cancer: a randomized trial. Eur Heart J. 2023;44(46):4878–89. doi:10.1093/eurheartj/ehad085. 36806405 PMC10702461

[28] Schmidt ME, Semik J, Habermann N, Cancer-related fatigue shows a stable association with diurnal cortisol dysregulation in breast cancer patients. Brain Behav Immun. 2016;52:98–105. doi:10.1016/j.bbi.2015.10.005. 26456694

[29] Kallich JD, Tchekmedyian NS, Damiano AM, Psychological outcomes associated with anemia-related fatigue in cancer patients. Oncology. 2002;16(9 Suppl 10):117–24. 12380961

[30] Mustian KM, Alfano CM, Heckler C, Comparison of pharmaceutical, psychological, and exercise treatments for cancer-related fatigue: a meta-analysis. JAMA Oncol. 2017;3(7):961–8. doi:10.1001/jamaoncol.2016.6914. 28253393 PMC5557289

[31] Chen X, Li J, Chen C, Effects of exercise interventions on cancer-related fatigue and quality of life among cancer patients: a meta-analysis. BMC Nurs. 2023;22(1):200. doi:10.1186/s12912-023-01363-0. 37312185 PMC10261838

[32] Mustian KM, Morrow GR, Carroll JK, Integrative nonpharmacologic behavioral interventions for the management of cancer-related fatigue. Oncologist. 2007;12(Suppl 1):52–67. doi:10.1634/theoncologist.12-S1-52. 17573456

[33] Mishra Y, Ranjan A, Mishra V, The role of the gut microbiome in gastrointestinal cancers. Cell Signal. 2024;115:111013. doi:10.1016/j.cellsig.2023.111013. 38113978

[34] Bullard BM, McDonald SJ, Cardaci TD, Nonpharmacological approaches for improving gut resilience to chemotherapy. Curr Opin Support Palliat Care. 2022;16(3):151–60. doi:10.1097/SPC.0000000000000599. 35862879

[35] Matei B, Winters-Stone KM, Raber J. Examining the mechanisms behind exercise’s multifaceted impacts on body composition, cognition, and the gut microbiome in cancer survivors: exploring the links to oxidative stress and inflammation. Antioxidants (Basel). 2023;12(7):1423. doi:10.3390/antiox12071423. 37507961 PMC10376047

[36] Estaki M, Pither J, Baumeister P, Cardiorespiratory fitness as a predictor of intestinal microbial diversity and distinct metagenomic functions. Microbiome. 2016;4(1):42. doi:10.1186/s40168-016-0189-7. 27502158 PMC4976518

[37] Wang B, Jin YX, Dong JL, Low-intensity exercise modulates gut microbiota to fight against radiation-induced gut toxicity in mouse models. Front Cell Dev Biol. 2021;9:706755. doi:10.3389/fcell.2021.706755. 34746120 PMC8566984

[38] Deng N, Reyes-Uribe L, Fahrmann JF, Exercise training reduces the inflammatory response and promotes intestinal mucosa-associated immunity in Lynch syndrome. Clin Cancer Res. 2023;29(21):4361–72. doi:10.1158/1078-0432.CCR-23-0088. 37724990 PMC10618653

[39] Carter SJ, Hunter GR, Blackston JW, Gut microbiota diversity is associated with cardiorespiratory fitness in post-primary treatment breast cancer survivors. Exp Physiol. 2019;104(4):529–39. doi:10.1113/EP087404. 30763983 PMC6464368

[40] Sougiannis AT, VanderVeen BN, Enos RT, Impact of 5 fluorouracil chemotherapy on gut inflammation, functional parameters, and gut microbiota. Brain Behav Immun. 2019;80:44–55. doi:10.1016/j.bbi.2019.02.020. 30807838 PMC6660349

